# Effectiveness of Exercise Programs on Patients with Dementia: A Systematic Review and Meta-Analysis of Randomized Controlled Trials

**DOI:** 10.1155/2019/2308475

**Published:** 2019-11-22

**Authors:** Xudong Li, Rui Guo, Zhenhong Wei, Jing Jia, Chaojun Wei

**Affiliations:** ^1^Department of Physical Education, Lanzhou University, Lanzhou, China; ^2^The Institute of Clinical Research and Translational Medicine, Gansu Provincial Hospital, Lanzhou, China

## Abstract

Exercise programs have been introduced to improve cognitive function, whereas studies showed inconsistent results regarding the effectiveness of exercise programs on patients with dementia. This study aimed to summarize randomized controlled trials (RCTs) to assess the effect of exercise programs on cognition, activities of daily living (ADL), and depression in elderly with dementia. We systematically screened PubMed, Embase, and the Cochrane library for relevant studies throughout November 21, 2018. The pooled standardized mean differences (SMDs) with 95% confidence intervals (CIs) were employed to calculate cognition, ADL, and depression by using random-effects model. A total of 20 RCTs with 2,051 dementia patients were included in final quantitative meta-analysis. There were no significant differences between exercise programs and control regarding cognition (SMD: 0.44; 95% CI: −0.21–1.09; *P*=0.183), ADL (SMD: 0.50; 95% CI: −0.03–1.02; *P*=0.066), and depression (SMD: −0.43; 95% CI: −0.90–0.05; *P*=0.077). Sensitivity analysis results indicated that exercise programs might play an important role in cognition and ADL, whereas the depression level was unaltered by the exclusion of any particular study. Subgroup analyses indicated that exercise programs were associated with increased cognitive levels if the mean age of patients was <80.0 years when compared with usual care and studies with low quality. Moreover, the ADL level was significantly increased in patients receiving exercise programs versus usual care. These results suggested that exercise programs might play an important role in cognition and ADL in patients with dementia. These results required further verification by large-scale RCTs, especially for depression outcomes.

## 1. Introduction

Dementia is a major neurological disorder that causes disability and dependency among individuals, and so it has become a significant global problem. The prevalence of dementia among the elderly (≥60 years) people is 4.86% worldwide [1]. The incidence of dementia is accompanied with the ageing process of the individuals, and its prevalence is increasing worldwide [2]. The characteristic of dementia involves a progressive declination in cognition, which in turn is associated with the loss of social and occupational functions [3]. Moreover, patients with dementia have poor balance and gait [4], and gait might be a surrogate marker of cognitive impairment and decline, which could affect the dependence of activities of daily living (ADL) [5]. Furthermore, there is convincing evidence that demonstrated that both notion and depression in early life are associated with increased risk of dementia in later life, while depression in later life could be regarded as a prodrome to dementia [6]. Although pharmacological treatments have been employed for improving cognitive function and ADL, various side effects and no disease modifications were observed in patients with dementia [7, 8]. Therefore, effective strategies should be explored for patients with dementia.

According to a previous study, exercise assists in gradually slowing down the progression of dementia. The potential reasons for this could be the fact that regular exercise has direct effects on the brain cortex, neuromuscular and cardiovascular functioning, immune system, arteriosclerosis in the brain, mood, and depression states [9]. Furthermore, regular exercises could decrease neuropathological burden and increase hippocampal neurogenesis [10, 11]. A previous systematic review was conducted on dementia patients and pointed out that regular exercise has no significant effect on cognition and depression, while it provides a beneficial effect on ADL [12]. However, this study evaluated the results of cognition, ADL, and depression based on the data after intervention, but the mean changes of these indexes were not calculated. Moreover, whether the treatment effects of exercise are differing according to patients' characteristics was not illustrated. Therefore, this current meta-analysis was conducted based on randomized controlled trials (RCTs) to determine the treatment effects of exercise programs on cognition, ADL, and depression in patients with dementia.

## 2. Materials and Methods

### 2.1. Data Sources, Search Strategy, and Selection Criteria

This study was conducted and reported according to the guidelines of Preferred Reporting Items for Systematic Reviews and Meta-Analysis Statement (PRISMA) [13]. The electronic databases of PubMed, Embase, and the Cochrane library were systematically searched for RCT studies published regarding the investigation of treatment effects of exercise programs in patients with dementia from their inception up to November 21, 2018. The following search terms as medical subjecting heading and free words were used: (exercise or training) and (dementia or Alzheimer's disease) and “English.” The detailed information regarding the search strategy was presented in [Supplementary-material supplementary-material-1]. The reference list of the retrieved studies was also reviewed to identify any new eligible study.

Two authors independently conducted literature search and study selection, and any inconsistencies between them were resolved by discussion with each other. The inclusion criteria of this study are as follows: (1) patients: patients without any restriction to age were diagnosed with dementia according to the diagnosis criteria in individual trial; (2) intervention: patients received regular exercise programs, and the details of exercise programs have been listed in Table 1; (3) control: control is patients with usual care and without regular exercise; (4) outcomes: the study should report at least 1 of the following outcomes: cognition, ADL, and depression; and (5) study design: study design is RCT design.

### 2.2. Data Collection and Quality Assessment

Data collection and quality assessment were carried out by 2 authors, and disagreement was adjudicated by an additional author by reading the full text of the article. The collected items included study, publication year, country, sample size, mean age, intervention, control, treatment duration, diagnostic criteria, and reported outcomes. The quality of included studies was evaluated by using the revised Jadad scale that is based on random sequence generation, allocation concealment, blinding, blinding of outcome assessment, incomplete outcome data, selective reporting, and other biases [34]. The “score system” for RCTs ranged from 0 to 7, where studies with score of 5 or more are regarded as high-quality studies.

### 2.3. Statistical Analysis

The treatment effects of exercise programs versus control on cognition, ADL, and depression based on mean, standard deviation, and sample size in each group in individual trial were calculated. The pooled standardized mean differences (SMDs) with 95% confidence intervals (CIs) were calculated for cognition, ADL, and depression using random-effects model [35, 36]. Heterogeneity was evaluated using *I*-square and *Q* statistic, and *P* < 0.10 was considered as significant heterogeneity [37, 38]. The stability of pooled results for investigating the outcomes was calculated by using sensitivity analyses [39]. Stratified analyses for cognition, ADL, and depression were performed based on publication year, country, sample size, mean age, control, treatment duration, and study quality. Univariable metaregression was conducted to evaluate the differences between subgroups [40]. The funnel plots, Egger et al. [41], and Begg and Mazumdar [42] tests were employed for evaluating the publication bias. The inspective levels for pooled results are 2-sided, and <0.05 was considered to be statistically significant. All analysis was conducted using STATA software (version 10.0; Stata Corporation, College Station, TX, USA).

## 3. Results

### 3.1. Search of the Published Literature

**Figure 1:**
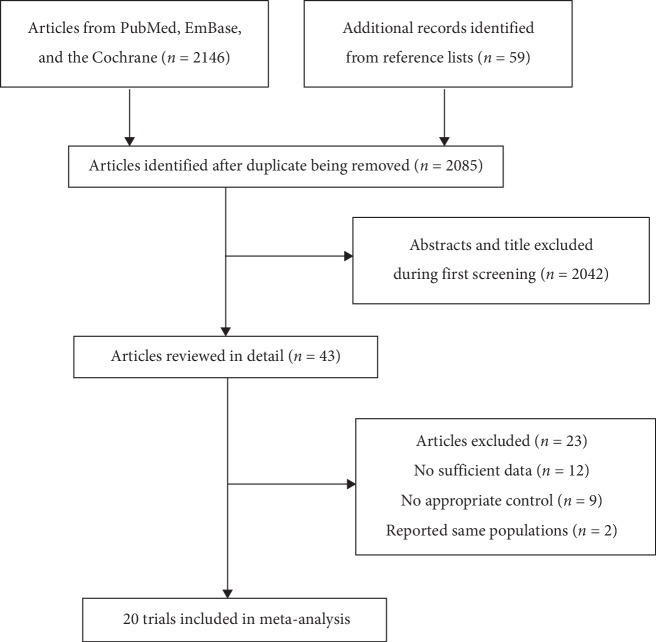
Flow diagram of literature search and trials selection process.

The electronic searches produced 2,146 records, and manual search of the reference lists of retrieved studies identified 59 studies. One hundred and twenty records were removed due to duplicate topics, and 2,042 studies were excluded due to irrelevant topics after studying the title and abstract. The remaining 43 studies were retrieved for full-text evaluations, and 23 studies of these were excluded due to the following reasons: no sufficient data (*n* = 12), no appropriate control (*n* = 9), and studies reporting similar populations (*n* = 2). Finally, 20 RCTs were identified for quantitative analysis [14–33]. The details of study selection process are shown in Figure 1. The baseline characteristics of included studies are presented in Table 1.

### 3.2. Study Characteristics

A total of 20 RCTs involving a total of 2,051 patients with dementia were included in the final analysis. The studies published between 1997 and 2018 and sample sizes ranged from 11 to 415 were included. The mean age of patients ranged from 70.5 to 87.9 years, and the treatment duration ranged from 6 weeks to 18 months. Fourteen studies were conducted in Europe, and the remaining 6 studies were conducted in USA, Brazil, Australia, Korea, and China. Eleven studies compared the exercise program with other strategies, while the remaining 9 studies compared the exercise program with usual care. The revised Jadad scale was used for quality evaluation, where 5 studies scored 6, 7 studies scored 5, 3 studies scored 4, 4 studies scored 3, and the remaining 1 study scored 2.

### 3.3. Cognition

**Figure 2:**
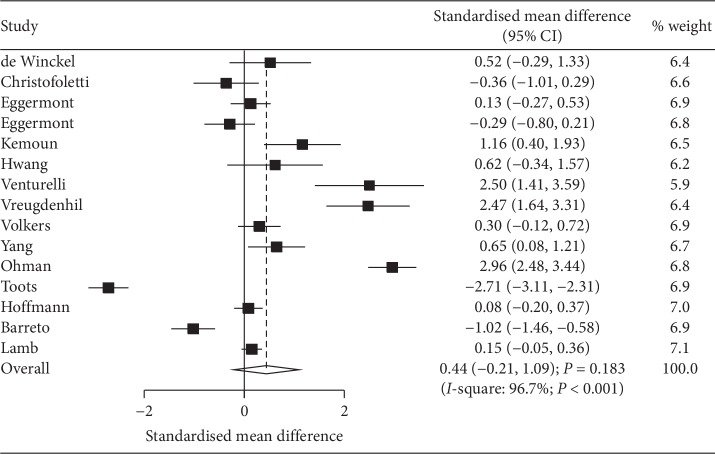
Effect of exercise programs on cognition.

Data regarding the effect of exercise program on cognition was available in 15 studies, and the pooled SMD indicated no significant differences between exercise program and control for cognition level (SMD: 0.44; 95% CI: −0.21 to 1.09; *P*=0.183; Figure 2). Moreover, substantial heterogeneity was observed among the included studies (*I*-square: 96.7%; *P* < 0.001). Sensitivity analysis indicated that exercise program had a beneficial effect on cognition after excluding the study conducted by Toots [29], and the study specified that receiving high-intensity functional exercise program aimed to improve lower limb strength, balance, and mobility ([Supplementary-material supplementary-material-1]). Subgroup analyses indicated that exercise program significantly improved cognition in patients with mean age of <80.0 years (SMD: 0.97; 95% CI: 0.07 to 1.87; *P*=0.035), compared with usual care (SMD: 1.06; 95% CI: 0.35 to 1.76; *P*=0.003) and pooled low-quality studies (SMD: 0.44; 95% CI: 0.06 to 0.83; *P*=0.024). No other significant differences were observed based on predefined factors (Table 2). Subgroup analysis indicated that the treatment effects of exercise program differed based on country (*P*=0.001), mean age (*P* < 0.001), control (*P* < 0.001), and study quality (*P*=0.006). Funnel plot did not rule out potential publication bias, and the Egger test (*P*=0.355) showed no significant publication bias, whereas the Begg test (*P*=0.048) showed potential publication bias ([Supplementary-material supplementary-material-1]).

### 3.4. ADL

**Figure 3:**
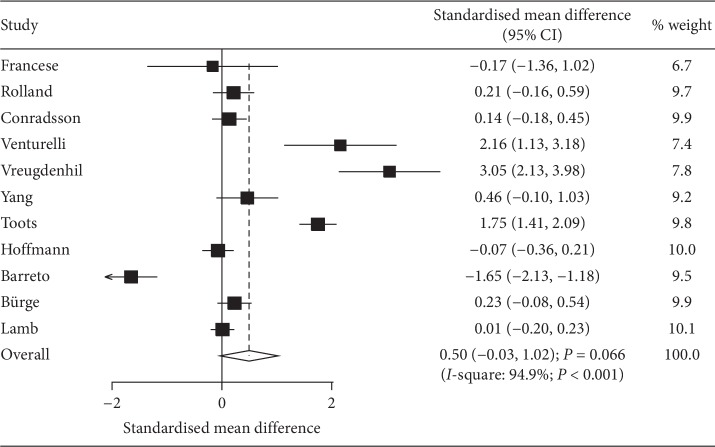
Effect of exercise programs on ADL.

Data regarding the effect of exercise program on ADL was available in 11 studies. We noted that exercise program has no significant effect on the levels of ADL when compared with control (SMD: 0.50; 95% CI: −0.03 to 1.02; *P*=0.066; Figure 3), whereas significant heterogeneity was observed (*I*-square: 94.9%; *P* < 0.001). Sensitivity analysis indicated that the conclusion was changed after excluding the trial conducted by de Souto Barreto et al. [31], which specifically compared music mediation or arts and crafts ([Supplementary-material supplementary-material-1]). Subgroup analysis indicated that exercise program significantly improved ADL when compared with usual care (SMD: 0.87; 95% CI: 0.19 to 1.54; *P*=0.012), and no other significant differences were detected (Table 2). Country (*P*=0.001), mean age (*P*=0.023), and treatment duration (*P* < 0.001) affected the exercise programs on ADL. No evidence of publication bias was observed (*P* value for Egger: 0.413; *P* value for Begg: 0.213; [Supplementary-material supplementary-material-1]).

### 3.5. Depression

**Figure 4:**
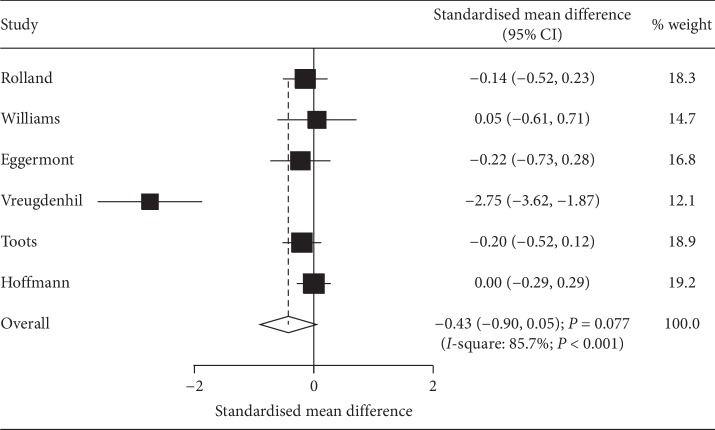
Effect of exercise programs on depression.

Data regarding the effect of exercise program on depression was available in 6 studies. The pooled SMD suggested that exercise programs did not yield any beneficial effects on depression level (SMD: −0.43; 95% CI: −0.90 to 0.05; *P*=0.077; Figure 4), and a significant heterogeneity among the included studies was detected (*I*-square: 85.7%; *P* < 0.001). Sensitivity analysis results indicated the stability of pooled conclusion after sequential exclusion of individual trial ([Supplementary-material supplementary-material-1]). Moreover, although the treatment effect of exercise program was affected by country (*P*=0.003) and sample size (*P*=0.022), no significant differences were observed between exercise programs and control for depression levels in all subsets (Table 2). There was no evidence of publication bias for depression (*P* value for Egger: 0.134; *P* value for Begg: 0.260; [Supplementary-material supplementary-material-1]).

## 4. Discussion

The current study was based on 2,051 patients with dementia from 20 RCTs with broad range of characteristics. Although significant heterogeneity was observed, we noted that the dementia patients who received exercise programs did not yield additional beneficial effects on cognition, ADL, and depression. Sensitivity analysis results indicated that exercise programs might play an important role in cognition and ADL. The beneficial effects of exercise programs on cognition were mainly observed in mean age of patients <80.0 years when compared with usual care and pooled low-quality studies. Moreover, we also noted that exercise programs could improve ADL when compared with usual care. These results are important for patients with dementia and warranted further large-scale RCTs to verify.

According to a previous systematic review based on 13 RCTs, AD patients receiving exercise programs showed positive effects on cognitive function, and 8/13 studies reported similar results, whereas the remaining 5 studies demonstrated no significant difference between exercise programs and control regarding cognitive function [43]. However, the results of ADL and depression are not reported, and stratified analyses based on patients' characteristics are not conducted. Blankevoort et al. indicated that physical activity has beneficial effects on patients with dementia during all stages. Moreover, combination of endurance, strength, and balance interventions significantly improved gait speed, functional mobility, and balance [44]. Lee et al. based on 9 studies suggested that dementia patients receiving physical capacity were associated with improved dementia symptoms, ADL, cognitive functions, and psychological state [45]. However, several important studies were not included in this study, and the treatment effects of exercise programs might be overestimated. Liang et al. conducted a network meta-analysis based on 17 RCTs and pointed out that physical exercise and computerized cognitive training play a beneficial role in cognition and neuropsychiatric symptoms in elderly patients with AD or mild cognitive impairment [46]. Due to these controversies, the current meta-analysis was conducted to demonstrate the treatment effects of exercise program versus control on cognition, ADL, and depression in patients with dementia.

Although the pooled SMD indicated no significant difference between exercise programs and control in cognition, this result was not stable and a beneficial effect might be observed. Five of the included studies reported similar positive results, whereas 2 trials reported opposite conclusion [29, 31]. Toots et al. indicated that high-intensity functional exercise program showed significant declination in ADL and improved balance in patients with non-Alzheimer's dementia, whereas exercise programs were associated with poor cognition [29]. The reason for this could be that the training programs focused on improving lower limb strength, balance, and mobility. de Souto Barreto et al. found that the decreased cognition level in exercise group was greater than that in patients who received music mediation or arts and crafts [31]. Patients recruited in this study were older than those in the other studies, and excess training might contribute additional burden in them, showing declination in cognitive function faster than expected. Subgroup analysis indicated that the treatment effect of exercise programs on cognition level mainly focused on mean age of patients of <80.0 years when compared with usual care and pooled low-quality studies. The potential reason for this could be that mean age was correlated with progression of dementia and the control strategy could affect the net treatment effect between exercise program and control. The quality of included studies could affect the evidence level, causing potential biases. The above results suggested that exercise programs were superior to usual care on cognition level, and the strategy of exercise programs should focus on strength and balance. Moreover, exercise programs should be given to younger patients with dementia.

Similarly, exercise programs did not yield additional beneficial effects on ADL, whereas a significant difference between exercise programs and control for ADL was observed. Most of the included studies reported no significant differences between exercise programs and control regarding the change in ADL, whereas de Souto Barreto et al. indicated that the levels of ADL in exercise group were lower than those in the control group [31]. Stratified analysis indicated that ADL was significantly improved in patients receiving exercise program when compared with usual care. This significant difference could be due to the fact that the net change between exercise and usual care was larger than the use of other strategies as control. In addition, there was no significant difference between exercise programs and control regarding the levels of depression. This effect was stable and unaltered by sensitivity and subgroup analyses. However, nearly all the included studies reported a positive trend in patients receiving exercise programs, which required further large-scale RCTs to demonstrate the treatment effects of exercise programs on depression.

However, our study has few limitations that should be mentioned. Firstly, the exercise strategy used by the included studies varied, and also the treatment effects of exercise programs differed. Secondly, the levels of cognition, ADL, and depression are evaluated by different scales in different studies, and substantial heterogeneity across the included studies was not fully interpreted. Thirdly, the type of dementia was not assigned in most of the included studies, and the analysis according to the types of dementia was not conducted. Fourthly, the summary results of depression were available in few studies and require verification in further studies. Fifthly, the analysis based on published studies and publication bias was inevitable. Finally, the detailed analysis was not conducted as this study used pooled data due to the unavailability of individual data.

## 5. Conclusion

In conclusion, these results suggested that exercise programs might play a beneficial role in cognition and ADL in patients with dementia, especially in younger patients and when compared with usual care, whereas exercise program showed no association with depression level. These results indicated that exercise programs should be introduced to patients with dementia, especially to younger patients. Further large-scale RCTs should be conducted to verify the treatment effects of exercise program on depression in patients with dementia.

## Figures and Tables

**Table 1:** Baseline characteristic of studies included in the systematic review and meta-analysis.

Study	Publication year	Country	Sample size	Mean age (years)	Percentage male	Setting	Intervention	Control	Treatment duration	Diagnosis criteria	Reported outcomes	Jadad scale
Francese et al. [14]	1997	USA	6/5	NA	NA	Nursing home	Exercises targeting strength and function that included the use of music, various types of exercise balls, and parachute leg weights	Social contact plus sing-along group that watched music videos	7 weeks	Clinical	ADL (CADS)	3

de Winckel et al. [15]	2004	Belgium	15/10	81.6	0.0	Public psychiatric hospital	Intervention focused on strength training, balance, trunk movements, and flexibility	Social contact 1-on-1 conversation with therapist	3 months	NIN CDS-ARDRA	Cognition (MMSE, ADS 6)	4

Rolland et al. [16]	2007	France	67/67	83.0	24.6	Nursing home	Aerobic (walking), strength (lower extremity), flexibility, and balance training, gradually increased in intensity	Usual care	12 months	NIN CDS-ARDRA	ADL (Katz index of ADLs), depression (MADRS)	6

Christofoletti et al. [17]	2008	Brazil	17/20	74.3	32.4	NA	Physiotherapy kinesiotherapy exercises (strength, balance, memory, and recognition exercise using balls, elastic ribbons, and proprioceptive plates)	Usual care	6 months	ICD-10, CMBD, and confirmed by the patient's performance on the MMSE and on KADL scale	Cognition (MMSE)	4

Williams and Tappen [18]	2008	USA	33/12	87.9	11.0	Nursing home	Exercise focusing on strength, flexibility, and balance; supervised walking	Social contact-conversation	16 weeks	NINCDS-ADRDA	Depression (CSDD)	4

Eggermont et al. [19]	2009	The Netherlands	51/46	85.4	18.6	Nursing home	Walking group, walks occurred on unit wards and in public places	Social contact	6 weeks	Clinical	Cognition (MMSE)	5

Eggermont et al. [20]	2009	The Netherlands	30/31	84.6	NA	Nursing home	Hand movement activity group performing activities such as “finger movement, pinching a soft ball, or handling a rubber ring”	Social contact plus read out loud program	6 weeks	DSM-IV	Cognition (RBMT), depression (GDS)	5

Conradsson et al. [21]	2010	Sweden	191	84.7	27.0	Nursing home	The high-intensity group exercise (3–9 participants per exercise group) focused on weight bearing and progressively increased in difficulty. Activity consisted of strength and balance exercises including walking, squats, and trunk exercises	Social contact plus seated activities provided by occupational therapists	13 weeks	KADL scale	ADL (Katz index of ADLs)	5

Kemoun et al. [22]	2010	France	20/18	81.9	21.1	Nursing home	The exercise program included three different sessions each week, i.e., (1) walking, (2) stamina exercise, and (3) a combination of walking, stamina, and balance exercises. For the first 2 weeks of the program, participants prepared for the routine program with specific muscles and joint exercises	Usual care	15 weeks	DSM-IV	Cognition (ERFC French version)	3

Hwang and Choi [23]	2010	Korea	10/8	81.5	NA	NA	A dance program consisting mainly of upper body exercises, with a 10-minute warm-up and warm-down	Usual care	8 weeks	Clinical	Cognition (MMSE)	2

Venturelli et al. [24]	2011	Italy	12/12	84.0	37.5	Nursing home	A minimum of 30 minutes of moderate walking 4 times a week for 6 months	Usual care at the home, which consisted of bingo, sewing, and music therapy	6 months	Clinical	Cognition (MMSE), ADL (Barthel index of ADL)	5

Vreugdenhil et al. [25]	2012	Australia	20/20	74.1	40.0	Outpatient memory disorders clinic	Exercises progressively became more challenging, and targeted strength and balance	Usual care	4 months	DSM-IV	Cognition (ADAS-cog), ADL (The instrumental ADL), depression (GDS)	6

Volkers [26]	2012	The Netherlands	50/38	82.1	NA	NA	Supervised walks	Usual care	18 months	Clinical	Cognition (MMSE)	3

Yang et al. [27]	2015	China	25/25	72.0	34.0	Neurology clinic	5 min warm-up, 30 min target intensity exercise, 5 min reorganization movement	Health education	3 months	NINDS-AIREN and MMSE	Cognition (MMSE, adas-cog), ADL (Qol-AD)	3

Ohman et al. [28]	2016	Finland	70/70	78.1	63.6	Community	Dual-task exercises, and strength, balance, endurance, and aerobic training; aerobic, endurance, balance, and strength training, and dual tasking	Usual care	12 months	NINCDS-ADRDA	Cognition (CDT, VF, CDR, MMASE)	5

Toots et al. [29]	2016	Sweden	93/93	85.1	24.2	Residential care facilities	High-intensity functional exercise program, which aims to improve lower limb strength, balance, and mobility	Seated control activity	7 months	DSM-IV-TR	Cognition (BBS), ADL (FIM and Barthel index of ADLs), depression (GDS)	6

Hoffmann et al. [30]	2016	Denmark	107/93	70.5	56.5	NA	The first four weeks of exercise (adaption) emphasized getting used to exercising and building up strength, primarily of the lower extremities (twice weekly). Participants were also introduced to aerobic exercise (once weekly). For the remaining 12 weeks, patients performed aerobic exercise of moderate-to-high intensity (in total 3 × 10 min on an ergometer bicycle, cross trainer, and treadmill with 2–5 min rest between)	Usual care	16 weeks	NINCDS-ADRDA	Cognition (SDMT), ADL (ADCS-ADL), depression (HAMD-17)	5

Barreto et al. [31]	2017	France	44/47	87.6	15.4	Nursing home	10 minutes of warm-up, 10 minutes of coordination and balance exercises, 10–15 minutes of muscle strengthening, 20 minutes of aerobic exercise, and 5–10 minutes of cool down	Music mediation or arts and crafts	24 weeks	DSM-IV and MMSE	Cognition (MMSE), ADL (ADCS-ADL-sev)	6

Bürge et al. [32]	2017	Switzerland	78/82	81.4	48.8	Psychiatric hospital	Squatting at different levels (or repeated stand-ups from a chair), lateral elevation of the legs in a standing position, and rising on the toes	Watching videos about different topics or playing together	6 weeks	CIM-10, and CDR	ADL (Barthel index of ADLs)	6

Lamb et al. [33]	2018	UK	278/137	77.3	60.7	National health service primary care, community and memory services	Arm exercises using hand held dumb bells, including at least a biceps curl and, for more able individuals, shoulder forward raise, lateral raise, or press exercises, and leg strength training exercises using a sit-to-stand weighted vest (all proexercise products, FL) or a waist belt (Rehabus, Lerum, Sweden), or both	Usual care	12 months	DSM-IV and MMSE	Cognition (ADAS-cog), ADL (Bristol ADL)	5

**Table 2:** Subgroup analyses for cognition, ADL, and depression.

Outcomes	Factors	Groups	SMD and 95% CI	*P* value	Heterogeneity (%)	*P* value for heterogeneity	*P* value between subgroups
Cognition	Publication year	Before 2010	−0.03 (−0.37 to 0.30)	0.845	31.4	0.224	0.366
		2010 or after	0.61 (−0.24 to 1.47)	0.160	97.6	<0.001	
	Country	Europe	0.31 (−0.47 to 1.08)	0.435	97.4	<0.001	0.001
		Others	0.83 (−0.28 to 1.93)	0.145	89.1	<0.001	
	Sample size	≥100	0.15 (−1.18 to 1.48)	0.825	98.8	<0.001	0.286
		<100	0.57 (−0.06 to 1.21)	0.076	90.2	<0.001	
	Mean age (years)	≥80.0	0.09 (−0.84 to 1.01)	0.854	96.1	<0.001	<0.001
		<80.0	0.97 (0.07 to 1.87)	0.035	96.6	<0.001	
	Control	Usual	1.06 (0.35 to 1.76)	0.003	95.1	<0.001	<0.001
		Others	−0.47 (−1.56 to 0.61)	0.395	96.6	<0.001	
	Treatment duration (months)	≥6	0.45 (−1.01 to 1.90)	0.548	98.6	<0.001	0.563
		<6	0.42 (−0.11 to 0.95)	0.123	88.9	<0.001	
	Study quality	High	0.43 (−0.51 to 1.38)	0.371	98.0	<0.001	0.006
		Low	0.44 (0.06 to 0.83)	0.024	51.2	0.069	

ADL	Publication year	Before 2010	0.18 (−0.18 to 0.54)	0.328	0.0	0.549	0.706
		2010 or after	0.59 (−0.02 to 1.21)	0.060	95.9	<0.001	
	Country	Europe	0.29 (−0.28 to 0.87)	0.317	95.6	<0.001	0.001
		Others	1.13 (−0.68 to 2.94)	0.223	92.3	<0.001	
	Sample size	≥100	0.37 (−0.13 to 0.88)	0.145	94.1	<0.001	0.068
		<100	0.75 (−0.98 to 2.48)	0.395	96.3	<0.001	
	Mean age (years)	≥80.0	0.43 (−0.44 to 1.31)	0.334	96.6	<0.001	0.023
		<80.0	0.57 (−0.08 to 1.23)	0.088	90.7	<0.001	
	Control	Usual	0.87 (0.19 to 1.54)	0.012	92.9	<0.001	0.080
		Others	0.14 (−0.76 to 1.04)	0.757	96.3	<0.001	
	Treatment duration (months)	≥6	0.97 (−0.01 to 1.95)	0.053	96.5	<0.001	<0.001
		<6	0.22 (−0.43 to 0.88)	0.504	93.6	<0.001	
	Study quality	High	0.56 (−0.04 to 1.15)	0.066	95.9	<0.001	0.681
		Low	0.35 (−0.16 to 0.86)	0.180	0.0	0.347	

Depression	Publication year	Before 2010	−0.13 (−0.41 to 0.14)	0.337	0.0	0.805	0.555
		2010 or after	−0.85 (−1.83 to 0.12)	0.085	94.2	<0.001	
	Country	Europe	−0.12 (−0.29 to 0.06)	0.191	0.0	0.778	0.003
		Others	−1.33 (−4.07 to 1.41)	0.341	96.0	<0.001	
	Sample size	≥100	−0.10 (−0.29 to 0.08)	0.285	0.0	0.640	0.022
		<100	−0.94 (−2.40 to 0.52)	0.209	93.1	<0.001	
	Mean age (years)	≥80.0	−0.16 (−0.37 to 0.05)	0.129	0.0	0.913	0.558
		<80.0	−1.34 (−4.03 to 1.35)	0.329	97.1	<0.001	
	Control	Usual	−0.85 (−1.90 to 0.19)	0.110	94.2	<0.001	0.757
		Others	−0.17 (−0.42 to 0.08)	0.186	0.0	0.773	
	Treatment duration (months)	≥6	−0.18 (−0.42 to 0.07)	0.159	0.0	0.823	0.797
		<6	−0.66 (−1.56 to 0.24)	0.153	91.4	<0.001	
	Study quality	High	−0.52 (−1.06 to 0.02)	0.058	88.4	<0.001	0.440
		Low	0.05 (−0.61 to 0.71)	0.876	—	—	

## Data Availability

The datasets used and/or analyzed during the current study are available from the corresponding author on reasonable request.

## Supplementary Materials

Supplementary Materials Supplement 1: searching strategy in PubMed, Embase, and Cochrane. Supplement 2: it includes 3 figures as follows. Figure S1: sensitivity analysis for cognition. Figure S2: sensitivity analysis for ADL. Figure S3: sensitivity analysis for depression. Supplement 3: it includes 3 figures as follows. Figure S1: funnel plot for cognition. Figure S2: funnel plot for ADL. Figure S3: funnel plot for depression.
