# Supplementary material for: Effectiveness of Exercise Programs on Patients with Dementia: A Systematic Review and Meta-Analysis of Randomized Controlled Trials
Source: Biomed Res Int. 2019 Nov 22;2019:2308475. doi: 10.1155/2019/2308475 (PMC6893254; doi:10.1155/2019/2308475)
Supplement: Supplementary Materials — Supplement 1: searching strategy in PubMed, Embase, and Cochrane. Supplement 2: it includes 3 figures as follows. Figure S1: sensitivity analysis for cognition. Figure S2: sensitivity analysis for ADL. Figure S3: sensitivity analysis for depression. Supplement 3: it includes 3 figures as follows. Figure S1: funnel plot for cognition. Figure S2: funnel plot for ADL. Figure S3: funnel plot for depression. [file 2308475.f1.zip › 2308475.f1/Supplemental 1.docx]

**Searching strategy in PubMed:**

1. exp Dementia/

2. Delirium/

3. Wernicke Encephalopathy/

4. Delirium, Dementia, Amnestic, Cognitive Disorders/

5. dement*.mp.

6. alzheimer*.mp.

7. (lewy* adj2 bod*).mp.

8. deliri*.mp.

9. (chronic adj2 cerebrovascular).mp.

10. (“organic brain disease” or “organic

brain syndrome”).mp

11. (“normal pressure hydrocephalus” and

“shunt*”).mp.

12. “benign senescent forgetfulness”.mp.

13. (cerebr* adj2 deteriorat*).mp.

14. (cerebral* adj2 insufficient*).mp.

15. (pick* adj2 disease).mp.

16. (creutzfeldt or jcd or cjd).mp.

17. huntington*.mp.

18. binswanger*.mp.

19. korsako*.mp.

20. or/1-19

21. exercis*.ti,ab.

22. physical activit*.ti,ab.

23. cycling.ti,ab.

24. swim*.ti,ab.

25. gym*.ti,ab.

26. (walk* or treadmill).ti,ab.

27. danc*.ti,ab.

28. yoga*.ti,ab.

29. “tai chi”.ti,ab.

30. exp Exercise/ or Exercise Therapy/

31. or/21-30

32. 20 and 31

33. randomized controlled trial.pt.

34. controlled clinical trial.pt.

35. randomi?ed.ab.

36. placebo.ab.

37. randomly.ab.

38. trial.ab.

39. groups.ab.

40. or/33-39

41. (animals not (humans and animals)).

sh.

42. 40 not 41

43. 32 and 42

44. (2011* or 2012*).ed.

45. 43 and 44

**Searching strategy in EmBase:**

1. exp dementia/

2. Lewy body/

3. delirium/

4. Wernicke encephalopathy/

5. cognitive defect/

6. dement*.mp.

7. alzheimer*.mp.

8. (lewy* adj2 bod*).mp.

9. deliri*.mp.

10. (chronic adj2 cerebrovascular).mp.

11. (“organic brain disease” or “organic

brain syndrome”).mp

12. “supranuclear palsy”.mp.

13. (“normal pressure hydrocephalus” and

“shunt*”).mp.

14. “benign senescent forgetfulness”.mp.

15. (cerebr* adj2 deteriorat*).mp.

16. (cerebral* adj2 insufficient*).mp.

17. (pick* adj2 disease).mp.

18. (creutzfeldt or jcd or cjd).mp.

19. huntington*.mp.

20. binswanger*.mp.

21. korsako*.mp.

22. CADASIL.mp.

23. or/1-22

24. exercis*.ti,ab.

25. physical activit*.ti,ab.

26. cycling.ti,ab.

27. swim*.ti,ab.

28. gym*.ti,ab.

29. (walk* or treadmill).ti,ab.

30. danc*.ti,ab.

31. yoga*.ti,ab.

32. “tai chi”.ti,ab.

33. exercise/ or stretching exercise/ or

anaerobic exercise/ or exercise intensity/ or

aerobic exercise/ or treadmill exercise/ or

aquatic exercise/

34. or/24-33

35. 23 and 34

36. randomized controlled trial/

37. controlled clinical trial/

38. randomi?ed.ab.

39. placebo.ab.

40. randomly.ab.

41. trial.ab.

42. groups.ab.

43. (“double-blind*” or “single-blind*”).ti,

ab.

44. or/36-43

45. 35 and 44

46. (2011* or 2012*).em.

47. 45 and 46

**Searching strategy in Cochrane:**

#1 MeSH descriptor Dementia explode all

trees

#2 MeSH descriptor Delirium, this term

only

#3 MeSH descriptor Wernicke Encephalopathy, this term only

#4 MeSH descriptor Delirium, Dementia,

Amnestic, Cognitive Disorders, this term

only

#5 dement*

#6 alzheimer*

#7 “lewy* bod*”

#8 deliri*

#9 “chronic cerebrovascular”

#10 “organic brain disease” or “organic

brain syndrome”

#11 “normal pressure hydrocephalus” and

“shunt*”

#12 “benign senescent forgetfulness”

#13 “cerebr* deteriorat*”

#14 “cerebral* insufficient*”

#15 “pick* disease”

#16 creutzfeldt or jcd or cjd

#17 huntington*

#18 binswanger*

#19 korsako*

#20 (#1 OR #2 OR #3 OR #4 OR #5 OR

#6 OR #7 OR #8 OR #9 OR #10 OR #

11 OR #12 OR #13 OR #14 OR #15 OR

#16 OR #17 OR #18 OR #19)

#21 MeSH descriptor Exercise explode all

trees

#22 exercis*

#23 “physical activit*”

#24 cycling

#25 swim*

#26 gym*

#27 walk* OR treadmill

#28 danc*

#29 yoga*

#30 “tai chi”

#31 aerobic*

#32 (#21 OR #22 OR #23 OR #24 OR #

25 OR #26 OR #27 OR #28 OR #29 OR

#30 OR #31)

#33 (#32 AND #20), from 2011 to 2012

[Trials]
