# Supplementary material for: Effectiveness of Exercise Programs on Patients with Dementia: A Systematic Review and Meta-Analysis of Randomized Controlled Trials
Source: Biomed Res Int. 2019 Nov 22;2019:2308475. doi: 10.1155/2019/2308475 (PMC6893254; doi:10.1155/2019/2308475)
Supplement: Supplementary Materials — Supplement 1: searching strategy in PubMed, Embase, and Cochrane. Supplement 2: it includes 3 figures as follows. Figure S1: sensitivity analysis for cognition. Figure S2: sensitivity analysis for ADL. Figure S3: sensitivity analysis for depression. Supplement 3: it includes 3 figures as follows. Figure S1: funnel plot for cognition. Figure S2: funnel plot for ADL. Figure S3: funnel plot for depression. [file 2308475.f1.zip › 2308475.f1/Supplemental 3.docx]

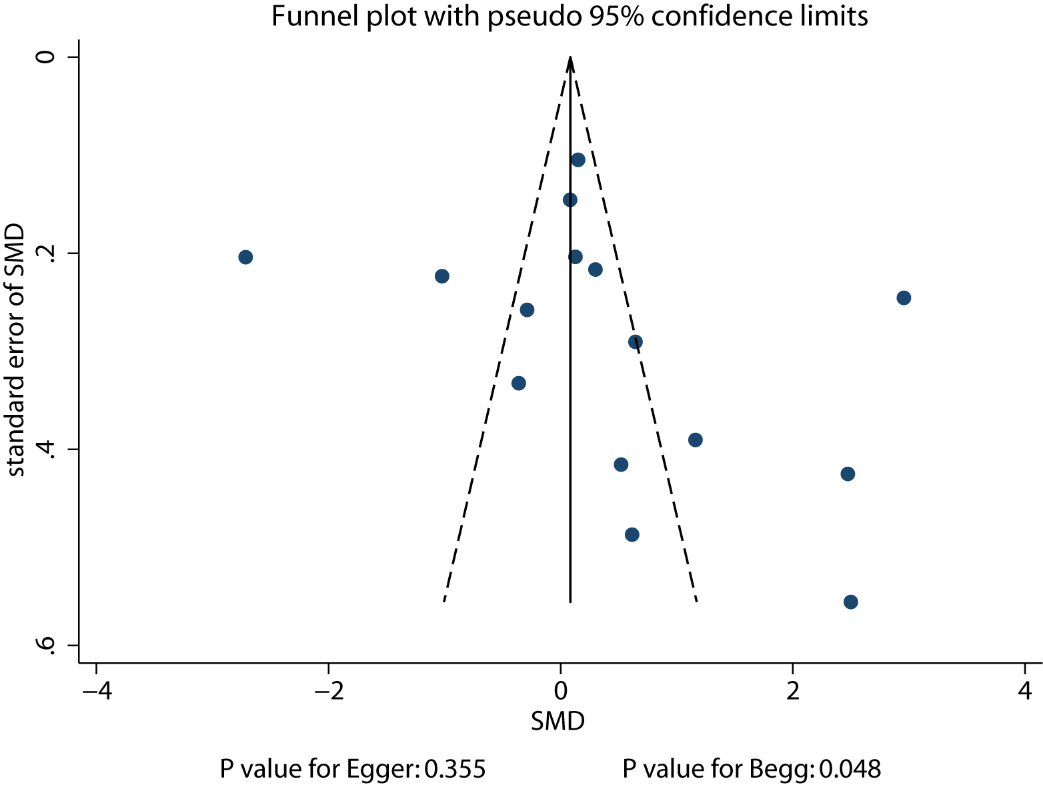


Figure S1. funnel plot for cognition


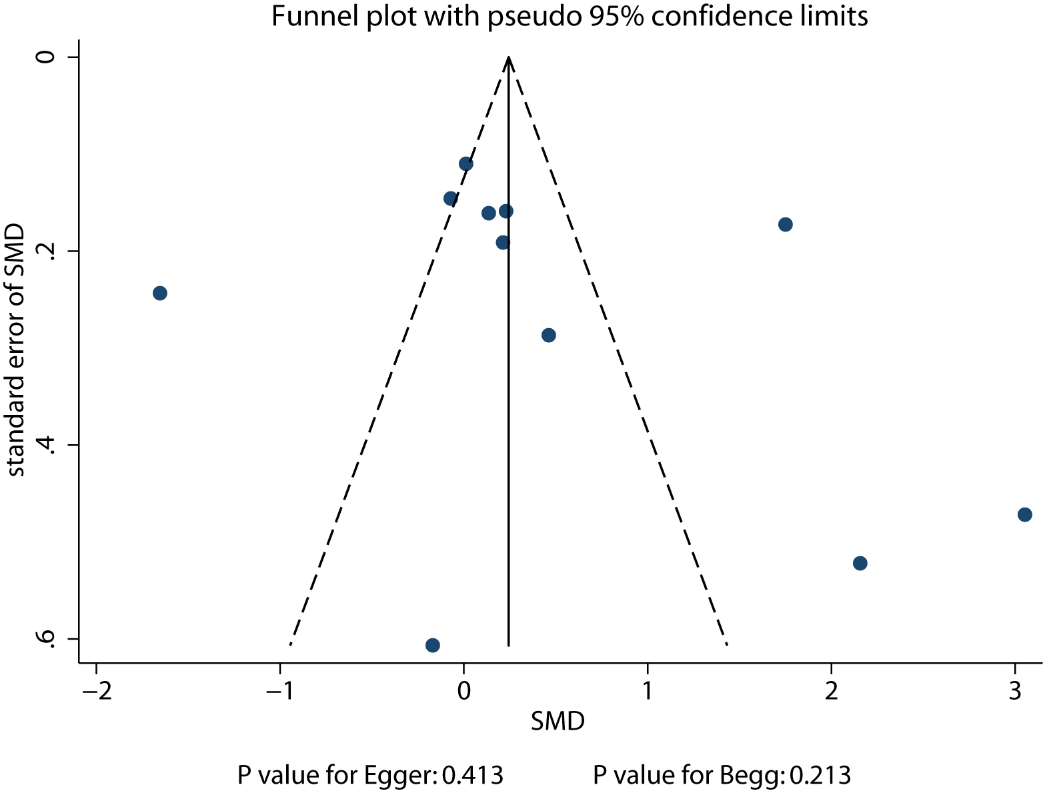


Figure S2. funnel plot for ADL


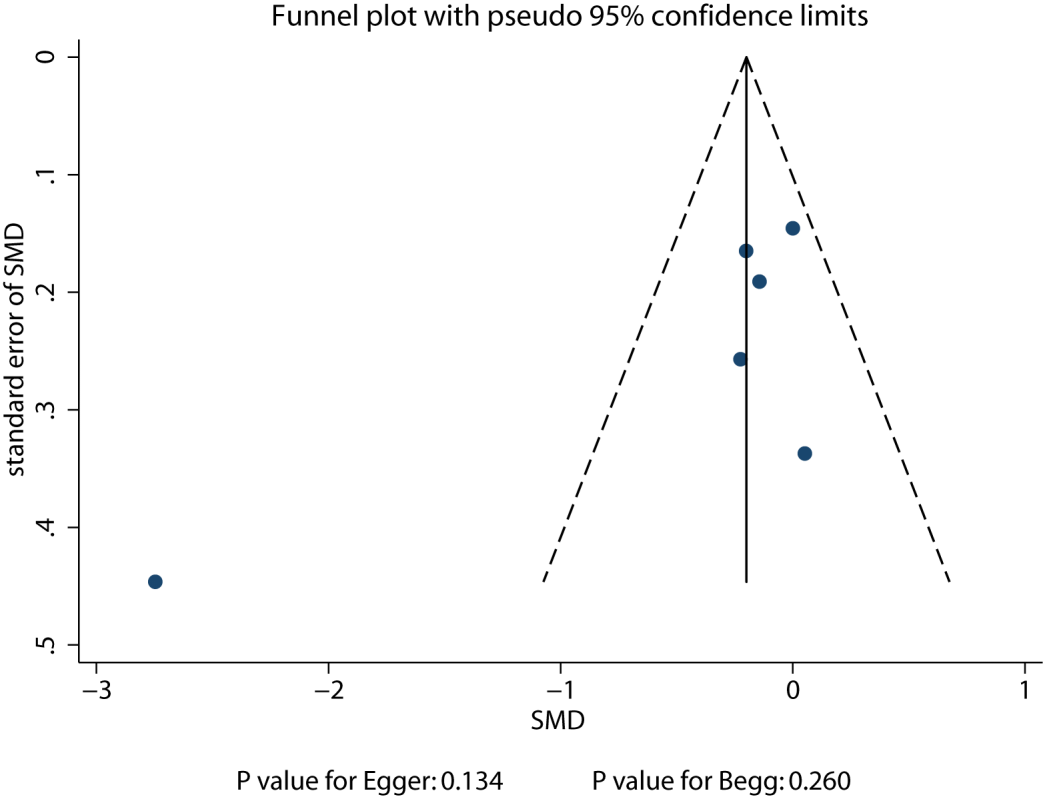


Figure S3. funnel plot for depression
